# Palmitic Acid Inhibits the Virulence Factors of *Candida tropicalis*: Biofilms, Cell Surface Hydrophobicity, Ergosterol Biosynthesis, and Enzymatic Activity

**DOI:** 10.3389/fmicb.2020.00864

**Published:** 2020-05-08

**Authors:** Krishnan Ganesh Prasath, Hariharan Tharani, Mourya Suraj Kumar, Shunmugiah Karutha Pandian

**Affiliations:** Department of Biotechnology, Alagappa University, Karaikudi, India

**Keywords:** *Candida tropicalis*, mature biofilm, palmitic acid, ROS, virulence factors

## Abstract

Biofilm is the fortitude of *Candida* species infections which eventually causes candidiasis in human. *C. tropicalis* is one of the predominant *Candida* species commonly found in systemic infections, next to *C. albicans*. In *Candida* species, biofilm maturity initiates irreversible surface attachment of cells and barricades the penetration of conventional antifungals. Hence, the current study investigated the antifungal and antivirulence potency of palmitic acid (PA) against *C. tropicalis* mature biofilm and its associated virulence factors. *In vitro* results revealed an effective inhibition of biofilm in PA-treated *C. tropicalis*, compared to *C. albicans* and *C. glabrata*. Also, PA reduced *C. tropicalis* mature biofilm at various time points. Further, PA treatment triggered apoptosis in *C. tropicalis* through ROS mediated mitochondrial dysfunction as demonstrated by confocal microscopic observation of PI, DAPI and DCFDA staining. PA regulated other virulence factors such as cell surface hydrophobicity, ergosterol biosynthesis, protease and lipase after 48 h of treatment. Downregulation of *ERG11* (Lanosterol 14-alpha demethylase) was contributed to the reduction of ergosterol in PA-treated *C. tropicalis*. However, enhanced hyphal growth was observed in PA-treated *C. tropicalis* through upregulation *HWP1* (Hyphal wall protein) and *EFG1* (Enhanced filamentous growth). This study highlighted the antibiofilm and antivirulence potency of PA against *C. tropicalis*. Hence, PA could be applied synergistically with other antifungal agents to increase the efficacy for regulating NCAC infections.

## Introduction

Clinically, non-C. albicans Candida (NCAC) species are increasingly reported as both colonizers and pathogenic in bloodstream infections. A multicenter study on candidemia epidemiology reported that the prevalence of *C. albicans* infection is higher in European nations (Poikonen et al., 2010; Asmundsdottir et al., 2012; Hesstvedt et al., 2015) but in case of United States (Cleveland et al., 2012), countries in Latin America (Nucci et al., 2013) and India (Chakrabarti et al., 2015), the occurrence of NCAC infections are higher than *C. albicans* infections. Among NCACs, *C. tropicalis* is the most commonly distributed compared to other species such as *C. glabrata*, *C. parapsilosis*, *C. krusei*, and *C. kefyr* (Kumari et al., 2014; Pahwa et al., 2014). A study at Indian rural tertiary hospital reported that the occurrence of *C. tropicalis* is phenomenal in urine, blood and oral scrapings from candidemia patients compared to *C. albicans* (Kaur et al., 2016). Also, *C. tropicalis* infection is found in surgical related infection such as osteomyelitis (Miller and Mejicano, 2001).

In our previous study, myristic acid from Nutmeg extract was shown to inhibit biofilm and hyphal formation by *C. albicans* by regulating proteins involved in sterol, sphingolipid and Multi-drug resistance pathways (Prasath et al., 2019). In addition, hexadecanoic acid was identified as a second major component through GC-MS analysis of nutmeg extract (Prasath et al., 2019). Hexadecanoic acid or Palmitic acid (PA), a saturated fatty acid is richly abundant in oil palms, meat, dairy products and many plants. PA possesses antimicrobial activity against numerous pathogens such as *Streptococcus mutants, Streptococcus gordonii, Streptococcus sanguis, C. albicans, Aggregatibacter actinomycetemcomitans* (Huang et al., 2011) but fails in *Propionibacterium acnes* (Yang et al., 2009).

Palmitic acid is one of the major components in cellular fatty acids of *Candida* species such as *C. parapsilosis, C. albicans, C. tropicalis*, and *C. famata* (Missoni et al., 2005). Also, PA is a product of Fatty acid Synthase (FAS) complex and is crucial for subsequent desaturation of fatty acid in *C. albicans* (Nguyen et al., 2009). PA at 2.5 mg mL^−1^ increases the cellular toxicity in *C. parapsilosis* and the rate of cell death is even higher in *ole1* (gene responsible for fatty acid desaturation) mutants by inducing Reactive oxygen species (ROS) (Nguyen and Nosanchuk, 2011). ROS are the aerobic by-product in both prokaryotes and eukaryotes during mitochondrial electron transport and metal catalyzed oxidation. The building up of ROS causes severe damage in cellular DNA, RNA and protein levels (Ray et al., 2012). Also, generation of ROS plays a vital role in altering virulence processes of the cell and most of the antifungal drugs induce ROS in both planktonic and biofilm cells (Delattin et al., 2014).

Similar to *C. albicans*, *C. tropicalis* is a dimorphic pathogen expressing a wide range of virulence factors such as biofilm, yeast-hyphal transition, hydrolytic enzymes and sterol synthesis. The expression level of these virulence factors are predominant during log growth phase. Also, the log-phase yeast cell resists external toxicity such as glucotoxicity by lipid storage mechanisms (Nguyen and Nosanchuk, 2011). The biofilm strengthens on excessive production of extracellular polymeric substance during late- log phase and forms mature biofilm (Montanaro et al., 2011). In *Candida* spp., the mature biofilm forms a complex structure and releases more daughter cells that disseminates to different niches to develop into new biofilms (Cavalheiro and Teixeira, 2018). During dual-biofilm formation, *C. albicans* suppresses the filamentation of *C. tropicalis* but the latter overpowers in biofilm formation during its association with *C. albicans* (Pathirana et al., 2019). Some conventional antibiotics holds potent antibiofilm activity on early-biofilm formation but fails to inhibit mature biofilm (Reiter et al., 2012). The antibiofilm activity of triazole drugs are not consistent and especially, fluconazole does not influence on the thickness of *C. albicans* biofilm (Chandra and Ghannoum, 2018). In this backdrop, the present study unveils the anti-infective potential of PA on mature biofilm and its associated virulence factors of *C. tropicalis* at different concentrations and time points.

## Materials and Methods

### *Candida* spp. Culture Conditions and Compound Concentration in This Study

1.2 × 10^5^ CFU mL^−1^ of *C. tropicalis* (MTCC 186), *C. albicans* (ATCC 90028), and *C. glabrata* (MTCC 3019) were cultured in YEPD medium (1% yeast extract, 2% peptone, 2% dextrose, Himedia Laboratories, Mumbai, India) by incubating at 37°C for 12 h at 160 rpm. Biofilm and dimorphism were analyzed by culturing 1.5 × 10^7^ CFU mL^−1^ of *Candida* spp. yeast cells in spider medium (1% mannitol, 0.25% K_2_HPO_4_, and 1% nutrient broth, Himedia Laboratories, Mumbai, India) and incubated for 48 h at 37°C. Palmitic acid (TCI chemicals, Japan) was dissolved in methanol as vehicle control with stock concentration of 10 mg mL^−1^.

### Determination of MIC of Palmitic Acid

The MIC of PA against *C. tropicalis* was determined using a macro broth dilution assay as per CLSI guidelines. Briefly, 3.2 × 10^7^ cells of *C. tropicalis* were allowed to grow in YEPD medium containing PA in a range of concentrations such as 2, 1, 0.5, 0.25, 0.125, 0.0625, 0.03125, and 0 mg mL^−1^ with 10 μg mL^−1^ of amphotericin B as positive control. The cells were incubated at 37°C for 10 h with agitation of 160 rpm. After incubation, cells were centrifuged and washed thrice with 1× PBS. The antifungal activity of PA was evaluated by measuring the absorbance of the cells at 600 nm (CLSI, 2017). In addition, the overnight grown *C. tropicalis* cells in the absence and presence of PA were serially diluted in PBS. Then, 3.1 × 10^5^ CFU mL^−1^ cells were spotted on YEPD agar medium followed by incubation at 37°C for 10 h. After incubation, the plates were documented in XR^+^ Bio-Rad gel doc system, United States.

### Effect of PA on *C. tropicalis* Biofilm Formation

The antibiofilm activity of PA was examined against the *C. tropicalis* at different concentrations such as 100, 200, 300, 400, 500, and 600 μg mL^−1^ in 24 well MTP. Briefly, 3.2 × 10^7^ cells were allowed to form biofilm in 24 well MTP in spider medium without and with PA and the plate was incubated at 37°C for 48 h. The biofilm cells were stained by 0.1% crystal violet (Sigma Aldrich, United States) followed by destaining with 10% glacial acetic acid. The absorbance of the solution was measured at 570 nm using Spectra Max 3 (Molecular Devices, United States). The percentage of biofilm inhibition was calculated using the formula,% Biofilm Inhibition = [(Absorbance of Control- Absorbance of Treated)/(Absorbance of Control)] × 100. In addition, antibiofilm activity of PA was also evaluated on *C. albicans* and *C. glabrata* biofilm formation.

### Determination of Biofilm Inhibitory Concentration of PA

In this experiment, PA at different concentrations (0, 25, 50, 75, 100, 125, 150, 175, 200, 225, and 250 μg mL^−1^) were added in spider medium in 24 well MTP. To that, 3.2 × 107 CFU mL^−1^ of *C. tropicalis* cells were inoculated and incubated at 37°C for 48 h. After incubation, the plate was stained with 0.1% crystal violet followed by destaining the plate with 10% glacial acetic acid. The absorbance of the destained solution was measured at 570 nm. The percentage of biofilm inhibition was calculated using the formula as mentioned above. The concentration of PA at which maximum percentage inhibition of *C. tropicalis* biofilm occurs, would be the BIC of PA. For growth assessment, the cells were harvested by centrifugation followed by washing twice with 1× PBS. The effect of PA on the growth of *C. tropicalis* was examined by spectrometric measurement of cell growth at 600 nm.

### Alamar Blue Assay

The effect of PA on the metabolism of *C. tropicalis* was determined by Resazurin, an oxidation-reduction indicator of cell viability. Briefly, Cells were allowed to grow in YEPD medium in the absence and presence of the PA at various concentrations as mentioned above in 96 well MTP for 12 h at 37°C. After incubation, 10 μg mL^−1^ of resazurin (Sigma Aldrich, United States) was added and incubated at 37°C for 4 h. The reduction of dye to pink color indicated the viability of cells and the reduction was measured in an excitation and emission wavelength of 560 nm and 590 nm, respectively (Selvaraj et al., 2019).

### Growth Kinetics of *C. tropicalis*

The effect of PA on *C. tropicalis* growth was analyzed by growth kinetics at different time points. Briefly, 3.2 × 10^7^ CFU mL^−1^ cells were used to inoculate in the presence and absence of PA at different concentrations such as 100, 200, 400, and 800 μg mL^−1^ at 37°C for 20 h. Also, 10 μg mL^−1^ of amphotericin B was used as a positive control. The absorbance of *C. tropicalis* in the absence and presence of PA was measured at 600 nm for every 2 h.

### Efficacy of the Compound on *C. tropicalis* Mature Biofilm

For mature biofilm study, 1.8 × 10^5^ CFU mL^−1^ *C. tropicalis* was used to inoculate in spider medium in 24 well MTP and incubated at various time points such as 1, 2, 4 and 7 days at 37°C. After the growth of *C. tropicalis* at the prescribed time points, the medium were removed and fresh spider medium along with PA (0, 100, 200, and 400 μg mL^−1^) were added. The plates were incubated on different time points such as 12, 24, and 48 h. After incubation, the planktonic cells were removed and the plates were stained with 0.1% crystal violet stain. Then, the stain was removed and washed with d.H_2_O. The plates were destained with 10% glacial acetic acid and the solution was quantified spectrophotometrically at 570 nm. The percentage biofilm inhibition of PA-treated cells was calculated by a formula as mentioned above.

### Light Microscopic Analysis of *C. tropicalis* Mature Biofilm

The effect of the compound on biofilm and hyphae formed by *C. tropicalis* was also observed using light microscopic analysis. Briefly, *C. tropicalis* was allowed to form biofilm in 1 × 1 cm sterile glass slides in spider medium in 24-well MTP and incubated at 37°C for 7 days. After incubation, PA at different concentrations such as 0, 100, 200, and 400 μg mL^−1^ were added and incubated at 37°C for 24 and 48 h. After incubation, the slides were washed with d.H_2_O to remove unbound cells and stained with crystal violet. *C. tropicalis* dimorphism and biofilm were visualized at 400× magnification by using binocular optical microscope and documented by Nikon Eclipse, Ti 100 digital camera.

### Evaluation of 3-D Biofilm Architecture by Confocal Microscopic Analysis

The inoculation of *C. tropicalis* and the treatment of PA were followed as stated above in 1 × 1 cm sterile glass slides in spider medium in 24 well MTP. Then, the slides were washed and stained with 0.1% of acridine orange (Sigma Aldrich, United States). After that, the biofilm construction was visualized using confocal laser scanning microscopy (CLSM) (Zeiss LSM 710, Carl Zeiss, Gottingen, Germany) by using argon laser with an excitation and emission filter of 488 nm and 500–640 nm, respectively (Gowrishankar et al., 2016). The thickness and 3-dimension visualization of biofilm construction were observed by image acquisition software (Zen 2009, Carl Zeiss).

### Colony Forming Unit Assay for Growth

The fungicidal effect of PA at different concentrations such as 0, 100, 200, and 400 μg mL^−1^ on *C. tropicalis* was examined in YEPD medium at 37°C for 24 and 48 h. After incubation, cells were washed thrice with 1× PBS. Then, the cells were subjected to serial dilutions followed by spreading on Sabouraud Dextrose Agar (SDA) and incubated at 37°C for 16 h. The colonies were counted in logarithmic scale and the plates were documented in Bio-Rad Gel Doc XR + System (United States).

### Scanning Electron Microscopy Analysis of Colony Morphology

The impact of PA on morphology of *C. tropicalis* was visualized in scanning electron microscope (SEM). *C. tropicalis* was allowed to grow in 1 × 1 cm glass slides in the absence and presence of PA for 48 h as mentioned above. The slides were washed and fixed with 2.5% glutaraldehyde for 2 h. Then, the slides were dehydrated with increasing concentration gradient of absolute ethanol from 50 to 100%. The dehydrated slides were visualized in SEM (Quanta FEG 250, United States) with the magnification of 2500×, 5000×, and 10000× (Prasath et al., 2019).

### Live and Dead Cell Analysis

The viability of *C. tropicalis* was also counter checked by live-dead cell analysis by using acridine orange (AO) and ethidium bromide (EtBr) stains. After treatment with PA at different concentrations (0, 100, 200, and 400 μg mL^−1^) for 24 and 48 h, the cells were stained with 1 mg mL^−1^ of AO and EtBr. The live and dead cells were visualized using CLSM with excitation and emission wavelength of 502 and 528 nm, respectively for AO and 470 and 600 nm for EtBr (Liu et al., 2015).

### Quantification of Apoptosis

The programed cell death was quantified by Propidium Iodide (PI- Sigma Aldrich, United States) staining solution which is permeable only in the dead cells. Briefly, the treated and untreated cells were collected at different time points such as 4 h, 8 h, 12 h, 24 h and 48 h. Then, the cells were washed with 1× PBS, stained with 50 μg mL^−1^ of PI solution and incubated in dark for 15 min. The apoptosis in untreated and treated cells was quantified by measuring the fluorescence intensity of PI with the excitation and emission wavelength of 535 and 617 nm, respectively (Crowley et al., 2016).

### Evaluation of DNA Damage

The DNA damage in *C. tropicalis* upon PA treatment was evaluated by 4’, 6-diamidino-2-phenylindole (DAPI - Sigma Aldrich, United States) staining. Briefly, *C. tropicalis* was treated in the absence and presence of PA at two different time points (24 and 48 h). After incubation, cells were harvested, stained with 2 μg mL^−1^ of DAPI and incubated in dark for 10 min. After the incubation, cells were washed with ice-cold PBS and the fluorescence intensity was visualized using CLSM with the excitation and emission wavelength of 358 and 461 nm, respectively (Rajavel et al., 2018).

### Detection of Reactive Oxygen Species

The apoptosis mediated by cellular reactive oxygen species (ROS) was studied using 2’,7’-dichlorodihydrofluorescein diacetate (DCFDA-Sigma Aldrich, United States) stain. ROS oxidize DCFDA to a fluorescence emitting DCF and the extent of fluorescence generated is directly proportional to the rate of ROS generated inside the cells. Briefly, untreated and PA-treated *C. tropicalis* cells at 24 and 48 h were stained with 15 μM DCFDA and incubated in dark for 1 h. After incubation, the cell suspension was centrifuged and excess staining solution was aspirated. Then, 500 μL of 1× PBS was added and the cell pellet was dispersed well in the solution. The difference in level ROS produced by untreated and treated cells at different time points were visualized in CLSM and quantified by florescence spectrophotometer with the excitation and emission wavelength of 488 and 530 nm, respectively (Shanmuganathan et al., 2019).

### Assessment of Mitochondrial Damage

The ROS mediated mitochondrial damage was assessed by measuring mitochondrial membrane potential (ΔΨ m) using Rhodamine 123 (Sigma Aldrich, United States) stain. Briefly, *C. tropicalis* cells were treated with PA at different concentrations (0, 100, 200, and 400 μg mL^−1^) and time points (24 and 48 h). After the incubation time, cells were fixed in 4% paraformaldehyde and stained with 10 μg mL^−1^ of Rhodamine 123. After incubation, fluorescence intensity was observed using CLSM and the images were documented (Wang and Youle, 2009).

### H_2_O_2_ Sensitivity Assay

*Candida tropicalis* cells grown for 24 and 48 h in the absence and presence of PA were adjusted to 0.3 of absorbance at 600 nm. Then, the cells were swabbed in YEPD agar plates and 10 mm filter paper discs (Himedia Laboratories, Mumbai, India) was mounted. Then the discs were loaded with 50 mM H_2_O_2_ and incubated at 37°C for 16 h. After incubation, the zone of clearance around the disc was measured and the plates were documented in Gel-doc system (Hassett et al., 1998).

### Evaluation of Superoxide Dismutase and Catalase by Native PAGE

The intracellular proteins of untreated and PA-treated *C. tropicalis* for 24 and 48 h were extracted by sonication. The extracted crude protein was separated in 8 and 10% native PAGE for catalase and SOD analysis, respectively. The gels were subjected to pre-run using running buffer (187.5 mM Tris and 1 mM EDTA) at 50 V for 20 min to eliminate excessive ammonium per sulfate. Then, the proteins were resolved in 50 mM Tris, 300 mM glycine and 1.8 mM EDTA running buffer at 60 V and 20°C. For catalase, gel was washed with 50 mM Potassium Phosphate buffer (pH 7) and incubated in 4 mM H_2_O_2_ for 10 min. After incubation, gel was stained with 1% potassium ferricyanide and 1% ferric chloride. The appearance of clear bands in the gel against dark green background indicated the presence of catalase activity of *C. tropicalis* (Sethupathy et al., 2016). For SOD, gel was stained in the 50 mM KPi (pH 7.8) buffer solution containing 0.1 mM EDTA, 2 mg riboflavin and 16 mg nitro blue tetrazolium (NBT). The reaction was initiated by adding 400 μL of Tetramethylethylenediamine (TEMED) to reduce NBT and incubated in dark for 1 h. After incubation, the gel was suspended in 50 mM KPi (pH 7.8) buffer solution followed by exposing the gels in bright light. The appearance of achromatic bands indicated the SOD activity of *C. tropicalis* (Prasath et al., 2019).

### Microbial Adhesion to Hydrocarbon Assay

The cell surface hydrophobicity (CSH) is the ability of cells that adhere to any hydrophobic substratum. The cells with greater hydrophobicity is directly correlated with the biofilm formation. CSH in *C. tropicalis* was determined through MATH assay as described by Sivasankar et al. (2015). Briefly, the treated and untreated cells were collected by centrifugation at 12000 rpm for 15 min. The cells were washed with 1× PBS thrice and the absorbance of cell suspension was measured at 600 nm. After that, 1 mL of toluene was added to the cell suspension and vortexed vigorously for 10 min. Then the solution was allowed to stand for phase separation. The organic phase was removed and the optical density (OD) of cell suspension in aqueous phase was measured spectrophotometrically at 600 nm. CSH was measured as hydrophobicity index using the following formula:

Hydrophobicityindex

$$\;=\left[1-\left(OD_{600\mathrm{fter}\mathrm{vortexing}}/OD_{600\mathrm{before}\mathrm{vortexing}}\right)\right]\times 100$$

### Lipase Assay

The effect of PA on *C. tropicalis* lipase was evaluated by both qualitative and quantitative method. For qualitative analysis, 3.5 × 10^5^ CFU mL^−1^ of untreated and treated *C. tropicalis* were spotted on the spider medium containing 0.1% of tributyrin as lipase substrate and incubated at 37°C for 5 days. After incubation, the zone of clearance around the cell growth indicates lipase activity of *C. tropicalis* (Muthamil et al., 2018). In addition, the lipase activity was measured quantitatively by mixing culture supernatant and 4-Nitro Phenyl palmitate (substrate) in the ratio 1:4. Then the solution was incubated in dark for 2 h and the absorbance of the solution was measured at 410 nm (Ramnath et al., 2017). The percentage of lipase inhibition was calculated using the following formula:

%ofLipaseInhibition

$$\;=[\left(\mathrm{Control}{\mathrm{OD}}_{410\mathrm{nm}}-{\mathrm{TreatedOD}}_{410\mathrm{nm}}\right)/$$

$$\;\left({\mathrm{ControlOD}}_{410\mathrm{nm}}\right)]\times 100$$

### Proteinase Assay

The extracellular proteinase produced by *C. tropicalis* was detected on Bovine Serum Albumin (BSA) agar medium containing dextrose – 2%, KH_2_PO_4 –_ 0.1%, MgSO_4_ – 0.05%, agar 2% and BSA 1% (Himedia Laboratories, Mumbai, India). Briefly, 3.5 × 10^5^ CFU mL^−1^ of untreated and PA-treated *C. tropicalis* was swabbed on BSA agar medium and incubated at 37°C for 7 days. The extracellular proteinase cleaves BSA and the zone of precipitation was observed around the *C. tropicalis* cells (Staib, 1965).

### Ergosterol Biosynthesis Assay

The untreated and PA-treated *C. tropicalis* cells for 24 and 48 h were harvested by centrifugation at 8000 rpm for 10 min and the cells were washed with 2 mL of 1× PBS thrice. Two mL of 20% alcoholic KOH was added to the cell pellet followed by incubation at 85°C for 1 h. Then, 1 mL of n-Heptane was added to the cell suspension and vortexed vigorously. After phase separation, the organic layer was transferred to a fresh tube and incubated at −20°C overnight. Absolute ethanol was added to the organic layer in the ratio 5:1 and the ergosterol content was measured spectrophotometrically in the range of 200–300 nm (Arthington-Skaggs et al., 1999).

### Filamentation Assay

The untreated and PA-treated *C. tropicalis* cells as mentioned above were adjusted to 0.3 of absorbance at 600 nm. Then, 3.5 × 10^5^ CFU mL^−1^ was spotted in spider agar medium and incubated at 37°C for 5 days. After incubation, the growth of hyphae on untreated and PA-treated (100, 200, and 400 μg mL^−1^) *C. tropicalis* was observed and the plates were documented in Bio-Rad Gel Doc XR + System (Liu et al., 1994).

### RNA Isolation and cDNA Conversion

Based on the virulence assays, the anti-infective potential of PA on virulence factors of *C. tropicalis* was validated by quantitative PCR (qPCR). RNA was isolated from untreated and PA-treated at 200 μg mL^−1^ for 48 h *C. tropicalis* cells by hot phenol method (Schmitt et al., 1990). The resulted RNA was observed by agarose gel electrophoresis and quantified by Nano spectrophotometer (Shimadzu, Japan) and 1 mg of total RNA was used for cDNA construction by Superscript III kit (Invitrogen Inc., United States).

### Virulence Gene Expression by Quantitative PCR

The cDNA and primers were added in the ratio 1:5 in a solution containing Quantinova SYBR Green PCR kit (Qiagen, Germany). The primers (Table 1) were designed by using Primer3 (Version: 0.4.0) software (Koressaar and Remm, 2007; Untergasser et al., 2012) and qPCR was performed in 7500 Sequence Detection System (Applied Biosystems Inc., Foster, CA, United States). The differential expression of virulence genes were normalized with β-actin of *C. tropicalis* and the relative fold change of gene expression levels were calculated using 2-^ΔΔ*CT*^ formula (2^−ΔΔ*CT*^ > 1- Upregulation and 2^−ΔΔ*CT*^ < 1- Downregulation of gene expression) (Prasath et al., 2019).

**TABLE 1:** List of virulent genes, functions and primer sequences used in qPCR study.

S. NO	GENE	PROTEIN	FUNCTION	PRIMER
1.	*ACT1*	Actin	Conserved protein involved in cell motility	F 5′- TTGCTCTCCGGCAACTTATT -3′ R 5′- TCGATCTTAATCGGGAGGTG3′
2.	*ERG11*	Lanosterol 14-alpha demethylase	Crucial step in ergosterol biosynthesis	F 5′- GAAAGAGAACCATTACCAGG -3′ R 5′- AGGAATCGACGGATCAC -3′
3.	*HWP1*	Hyphal wall protein	Hyphal cell wall protein plays role in hyphal developement	F 5′- CCCAGAAAGTTCTGTCCCAGT- 3′ R 5′- CCAGCAGGAATTGTTTCCAT -3′
4.	*TUP1*	Negative regulator of transcription	Represses *HWP1* for initiating filamentous growth	F 5′ -TTGCACCAGTTTCTGCAGTC-3′ R 5′- TTCAGCACCAGTAGCCAAGA -3′
5.	*NRG1*	Negative regulator of transcription	Regulation of chlamydospore formation, hyphal growth	F 5′- CCAAGTACCTCCACCAGCAT-3′ R 5′- GGGAGTTGGCCAGTAAATCA-3′
6.	*UME6*	Positive regulator of transcription	Promotes hyphal elongation	F 5′- ACCACCACTACCACCACCAC -3′ R 5′- TATCCCCATTTCCAAGTCCA - 3′
7.	*EFG1*	Enhanced filamentous growth protein 1	Important for filamentous growth	F 5′- GCCTCGAGCACTTCCACTGT R 5′- TTTTTTCATCTTCCCACATGGTAGT-3′

### Statistical Analysis

Power and sample sizes were calculated using G^∗^Power (UCLA, Los Angels, United States) (Faul et al., 2007). At 0.05 level of significance, the application of the one-way analysis of variance (ANOVA) *F*-test would have a power of 80.72% with sample size of 24 (*n* = 6 per group) and medium effect size value (difference of the means between the lowest and the highest value) of 0.75.

All the experiments were performed in three biological replicates with experimental triplicates. The data were presented as mean ± standard deviation. The mean differences between control and treated samples were analyzed statistically by one-way ANOVA and Duncan’s *post hoc* test was performed with the significant *p*-value of < 0.05, < 0.01, and < 0.001 using SPSS statistical software, version 17.0, United States. The symbols “✽”, “✯”and “
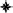
” denoted statistical significance with *p* < 0.05, < 0.01, and < 0.001, respectively.

## Results

### Antifungal Activity of PA Against *C. tropicalis*

**FIGURE 1:**
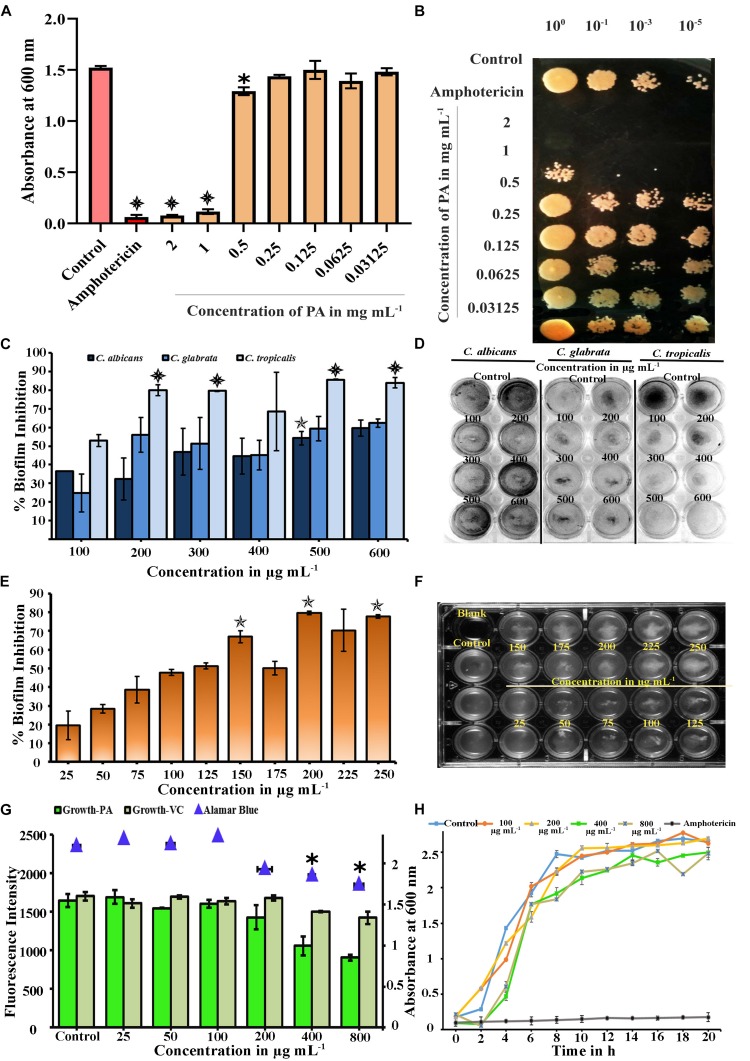
**(A)** Microbial Inhibitory Concentration (MIC) of PA against *C. tropicalis* was determined by Broth Dilution assay. PA reduced visible growth of *C. tropicalis* at 2 (*p* < 0.001) and 1 mg mL^−1^ (*p* < 0.001) and antifungal effect was compared with 10 μg mL^−1^ of amphotericin (*p* < 0.001). **(B)** Effect of PA on growth of Control and PA-treated *C. tropicalis* after serial dilution and spotting in YEPD agar medium with amphotericin B as positive control **(C)** Effect of palmitic acid (PA) on *Candida* species biofilm grown in spider medium. PA decreased the biofilm at 200, 300, 500, and 600 μg mL^−1^ (*p* < 0.001) in *C. tropicalis* and did not inhibit significantly in *C. albicans* and *C. glabrata*. **(D)** Influence of PA on *Candida* species biofilm stained with CV, a representative image. **(E)** Inhibitory effect of PA on *C. tropicalis* biofilm and maximum inhibition was at 200 μg mL^−1^ (*p* < 0.01). **(F)** Representative MTP plate showing biofilm disruption ability of PA. **(G)** Difference between the impact of PA and its vehicle control on *C. tropicalis* growth and metabolic activity at different concentrations (25–800 μg mL^−1^). **(H)** Growth curve analysis of *C. tropicalis* with absence and presence of PA (100, 200, 400, and 800 μg mL^−1^) and 10 μg mL^−1^ of amphotericin B as positive control. The values are expressed as means and error bars depict standard deviation (*n* = 6). Significant values were determined by one-way ANOVA in terms of *p*-value (“✽”, “✯”, and “
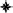
” denote *p* < 0.05, *p* < 0.01, and *p* < 0.001, respectively) followed by Duncan *post hoc* test.

Plants are the known source of novel bioactives with different functional groups such as phenolic, quinones, flavonoids and saponins, which possess antifungal activity (Arif et al., 2009). In this experiment, the effect of PA (2–0.03125 mg mL^−1^) on the growth of *C. tropicalis* was evaluated by broth dilution and spot assays. At lower concentrations, PA (at concentration range of 0.03125–0.25 mg mL^−1^) did not affect the growth of *C. tropicalis.* Beyond that, PA reduced the growth at 0.5 mg mL^−1^ with *p* < 0.05. Further, PA at 1 and 2 mg mL^−1^ prevented the visible growth of *C. tropicalis* significantly with *p* < 0.001 (Figure 1A). The antifungal activity of PA was corroborated with the amphotericin B (*p* < 0.001) as positive control. Contemporarily, the antifungal activity of PA was counterchecked with spot assay (Figure 1B), which reflected the same as in broth dilution method. Both studies revealed that PA at 1 mg mL^−1^ was found to be MIC against *C. tropicalis*.

### Effect of PA on *Candida* Species Biofilm Formation

The broth dilution assay revealed that PA was antifungal in the range of 500 μg mL^−1^. Hence, the antibiofilm activity of PA at different concentrations ranges from 100 to 600 μg mL^−1^ were tested against various *Candida* species such as *C. albicans, C. tropicalis* and *C. glabrata*. In *C. albicans* and *C. glabrata*, biofilm inhibition was observed PA-treated cells at 500 μg mL^−1^ with percentage inhibition of 54.22% (*p* < 0.01) and 59.37%, respectively. But PA effectively inhibited 52.98% of biofilm formed by *C. tropicalis* at 100 μg mL^−1^ (Figures 1C,D). Also, significant reduction of biofilm was observed in PA-treated *C. tropicalis* at 200, 500, and 600 μg mL^−1^ with percentage inhibition of 80.02% (*p* < 0.001), 85.64% (*p* < 0.001), and 84.03% (*p* < 0.001). From this study, it is apparent that the antibiofilm activity of PA was higher in *C. tropicalis* than other *Candida* species.

### Effect of PA on *C. tropicalis* Biofilm in Dose-Dependent Concentrations

The effect of PA was evaluated on *C. tropicalis* biofilm at dose-dependent concentrations such as 25, 50, 75, 100, 125, 150, 175, 200, 225, 250 μg mL^−1^. *C. tropicalis* biofilm was inhibited with an increasing concentration of PA as shown in Figure 1E. Besides that, the biofilm inhibition of *C. tropicalis* was higher at 150 μg mL^−1^ (66.91%) with *p* < 0.01 and maximum inhibition was observed at 200 μg mL^−1^ (79.62%) with *p* < 0.01. Also, the cell accumulation on the medium surface was increased with increasing concentration of PA (Figure 1F). This proved that PA possessed the ability to disrupt the *C. tropicalis* biofilm with minimum biofilm inhibitory concentration (mBIC) at 200 μg mL^−1^.

### PA Impacted on *C. tropicalis* Growth

The effect of PA on the growth of *C. tropicalis* was evaluated by spectrophotometric method and the concentrations (W/V) of PA were compared with the corresponding concentrations (V/V) of vehicle control (methanol). Compared to control cells, the growth was intact in PA-treated *C. tropicalis* at 25, 50, 100, and 200 μg mL^−1^. The growth was reduced significantly at 400 and 800 μg mL^−1^ PA-treated *C. tropicalis*. However, vehicle control did not have any substantial effect even at higher concentrations (Figure 1G). Hence, PA with increasing concentrations regulated biofilm and growth of *C. tropicalis* without any influence of vehicle control.

### Assessment of Metabolic Activity of *C. tropicalis* Using Alamar Blue

In healthy cells, reduction of resazurin to pink colored resorufin by mitochondrial dehydrogenase, signifies the viability of cellular metabolism. The absorbance of the reduced substrate in untreated and PA-treated *C. tropicalis* was measured at 570 nm. Compared to control, metabolic activity of cells was not reduced until 0.5× BIC of PA. The metabolic activity was reduced significantly in PA-treated *C. tropicalis* at 400 (*p* < 0.05) and 800 μg mL^−1^ (*p* < 0.05) (Figure 1G).

### Effect of PA on *C. tropicalis* Growth Kinetics

Spectrometric analysis and metabolic assay demonstrated that BIC of PA reduced the growth of *C. tropicalis* and were counter examined by growth curve analysis. The cell growth was stable in both control and 100 μg mL^−1^ PA-treated cells with slight deterioration at 200 μg mL^−1^ PA-treated cells. At 400 and 800 μg mL^−1^, the growth of *C. tropicalis* was fungistatic and the antifungal activity was compared with the 10 μg mL^−1^ of amphotericin B (Figure 1H). Our previous experiments showed that PA was effective on *C. tropicalis* biofilm with impact on the cell growth. Moreover, the antibiofilm activity of PA at 400 and 800 μg mL^−1^ treatment was found to be analogous. Hence, the concentrations of PA was narrowed down to 400 μg mL^−1^ for further experiments.

### Time Killing Assay for *C. tropicalis* Mature Biofilm

**FIGURE 2:**
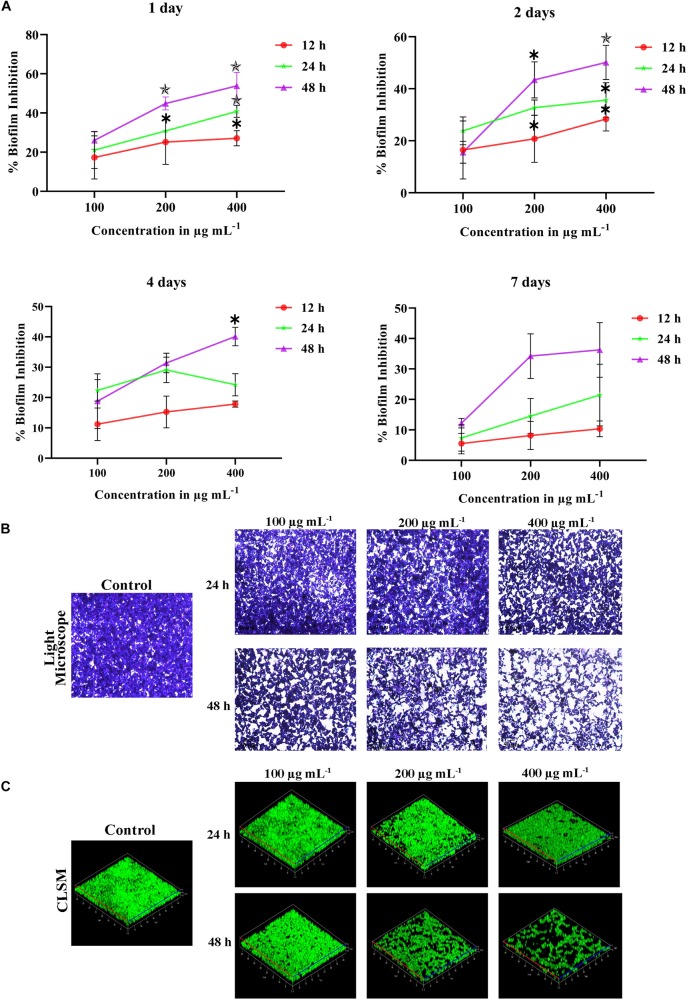
**(A)** Time killing of 1, 2, 4 and 7 days matured biofilm formed by *C. tropicalis* by PA at different time points (12, 24, and 48 h) and concentrations (100, 200, and 400 μg mL^−1^. The values are expressed as means and error bars indicate standard deviation of triplicates (*n* = 6). Control and PA-treated for 24 h and 48 h *C. tropicalis* biofilm (7 days matured) formed on 10 × 10 mm glass surfaces, as observed under **(B)** optical (Magnification – 400 ×, Scale bar – 50 μm) and **(C)** Confocal Microscope. ^✽^*p* < 0.05, ^✯^*p* < 0.01, and 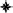
*p* < 0.001, significantly different when compared with control.

Some antibiofilm agents are effective on reducing early biofilm but not good enough against the mature biofilm. Farnesol, a quorum sensing molecule of *C. albicans*, inhibits germination of blastospores and biofilm but fails on mature biofilm (Blankenship and Mitchell, 2006). In our study, the effect of PA at different concentrations such as 100, 200, and 400 μg mL^−1^ were evaluated on 1, 2, 4 and 7 days matured biofilm with treatment time of PA on 12, 24 and 48 h (Figure 2A). On 1 day matured biofilm, PA at 200 μg mL^−1^ disrupted 30.84% with *p* < 0.05 after 24 h of treatment. Further, the efficacy of PA at 200 and 400 μg mL^−1^ was increased with 44.85% (*p* < 0.01) and 53.84% (*p* < 0.01), respectively after 48 h of treatment. Similar effect of PA was observed in 2 days matured *C. tropicalis* biofilm with 43.40% (*p* < 0.01) and 50.10% (*p* < 0.01) of inhibition upon 48 h of PA-treatment at 200 and 400 μg mL^−1^, respectively. On 4 and 7 days matured biofilm, the effect of PA (100, 200, and 400 μg mL^−1^) was moderated upon 12 and 24 h of treatment. But in case of 48 h treatment, PA at 200 μg mL^−1^ inhibited 31.38% and 34.20% of 4 and 7 days matured biofilm, respectively. At 400 μg mL^−1^ of PA, 4 and 7 days matured biofilm was reduced further with 40.71% (*p* < 0.05) and 36.25%.

### Light Microscopic Analysis of Mature Biofilm

The effect of PA on *C. tropicalis* 7 days matured biofilm was examined in optical microscope with 200 x magnification. Results showed that the mature biofilm formation was disturbed in a concentration-dependent manner. Upon 24 h of treatment, significant inhibition of biofilm was found in PA-treated *C. tropicalis* at 200 μg mL^−1^. On the other hand, a notable disruption in biofilm was observed beyond 100 μg mL^−1^ and the disruption was increased at 200 and 400 μg mL^−1^ of PA after 48 h treatment (Figure 2B).

### PA Reduced Biofilm Thickness and Density

The biofilm thickness and density formed by *C. tropicalis* was assessed by Z-stack of CLSM. The untreated *C. tropicalis* cells were enhanced with both hyphal and yeast cells (Figure 2C). Both biofilm thickness and density were reduced in the presence of PA with increasing concentrations on both 24 and 48 h treatment. But hyphal cells were observed even at higher concentration of PA upon treatment on both the time points.

### Time-Dependent Assessment of *C. tropicalis* Growth on PA Treatment

**FIGURE 3:**
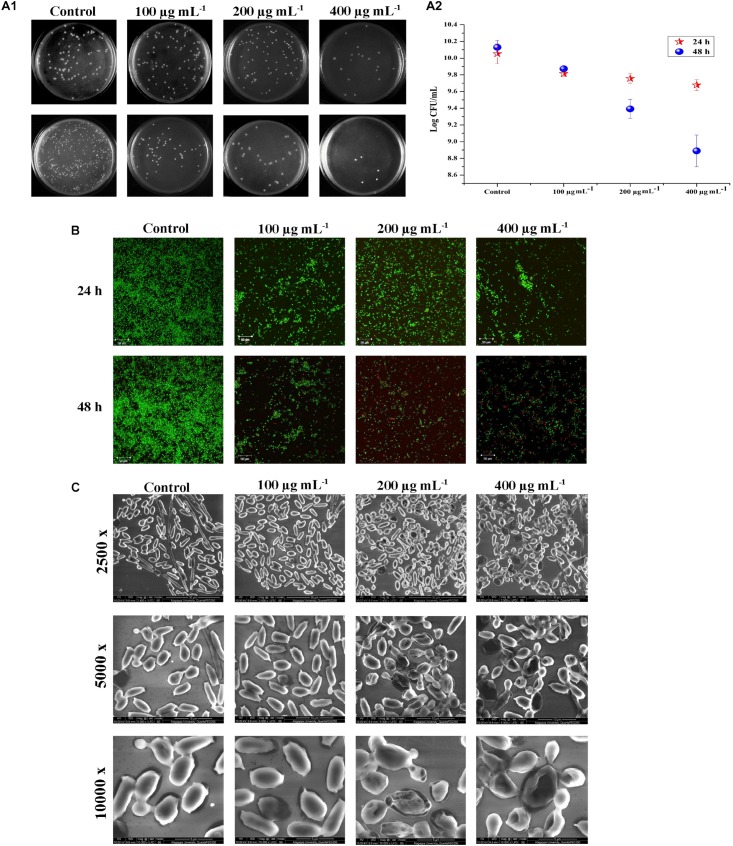
Effect of PA (0, 100, 200, and 400 μg mL^−1^) on *C. tropicalis* survival after 24 and 48 h of treatment. **[A (i)]** Image displaying the effect of PA on *C. tropicalis* survival, grown on SDA agar medium. **[A (ii)]** CFU of *C. tropicalis* in the absence and presence of PA determined in logarithmic scale. **(B)** Confocal microscopic examination of *C. tropicalis* revealing the efficacy of inducing cell death which was assessed using AO/EtBr staining. **(C)** SEM analysis of *C. tropicalis* colony morphology in the absence and presence of PA (100, 200, and 400 μg mL^−1^) after 24 and 48 h of treatment.

Biofilm and the associated virulence factors proliferate on late-log phase of *C. tropicalis.* The effect of PA upon 24 and 48 h treatment on 10 h grown *C. tropicalis* was examined by CFU method. After treatment with PA, the cells were serially diluted and spread on SDA agar medium. On 24 h treatment of PA, *C. tropicalis* colonies got reduced at 400 μg mL^−1^ compared to control. But after 48 h treatment, the colony reduction was seen even at 100 μg mL^−1^ and reduced further with increasing concentration of PA (Figure 3A (i)). The logarithmic conversion of untreated and PA-treated *C. tropicalis* CFU is shown in Figure 3A (ii).

### Testing Viability of *C. tropicalis* by Live – Dead Staining

Acridine orange (AO) is a cell-membrane permissible dye, whereas ethidium bromide (EtBr) cannot penetrate the live cells. Post treatment of PA at different time points on viability of *C. tropicalis* were assessed by staining the cells with AO and EtBr. Compared to control, dead cells were not seen in 100 μg mL^−1^, whereas countable dead cells were present at 200 and 400 μg mL^−1^ PA-treated cells upon 24 h. Further on 48 h of treatment, PA (200 and 400 μg mL^−1^) increased the membrane permeability of *C. tropicalis* with increasing concentrations that reflects higher number of dead cells (Figure 3B).

### SEM Analysis of *C. tropicalis* Cellular Morphology

As shown in CFU assay and Live-Dead staining, PA reduced the cellular growth of *C. tropicalis* after 48 h of treatment. Hence SEM was used to analyze the colony morphology of 48 h PA-treated *C. tropicalis* (Figure 3C). The untreated *C. tropicalis* composed of intact hyphae and yeast cells. PA at 100 μg ml^−1^ began to impose stress on cellular surface. At higher concentrations (200 and 400 μg mL^−1^) of PA, stress was surged and damage cells were observed. Here, SEM analysis proved that PA is fungistatic by mediating stress condition on *C. tropicalis* at higher concentrations upon 48 h of treatment.

### Quantification of Programed Cell Death by PA in *C. tropicalis*

**FIGURE 4:**
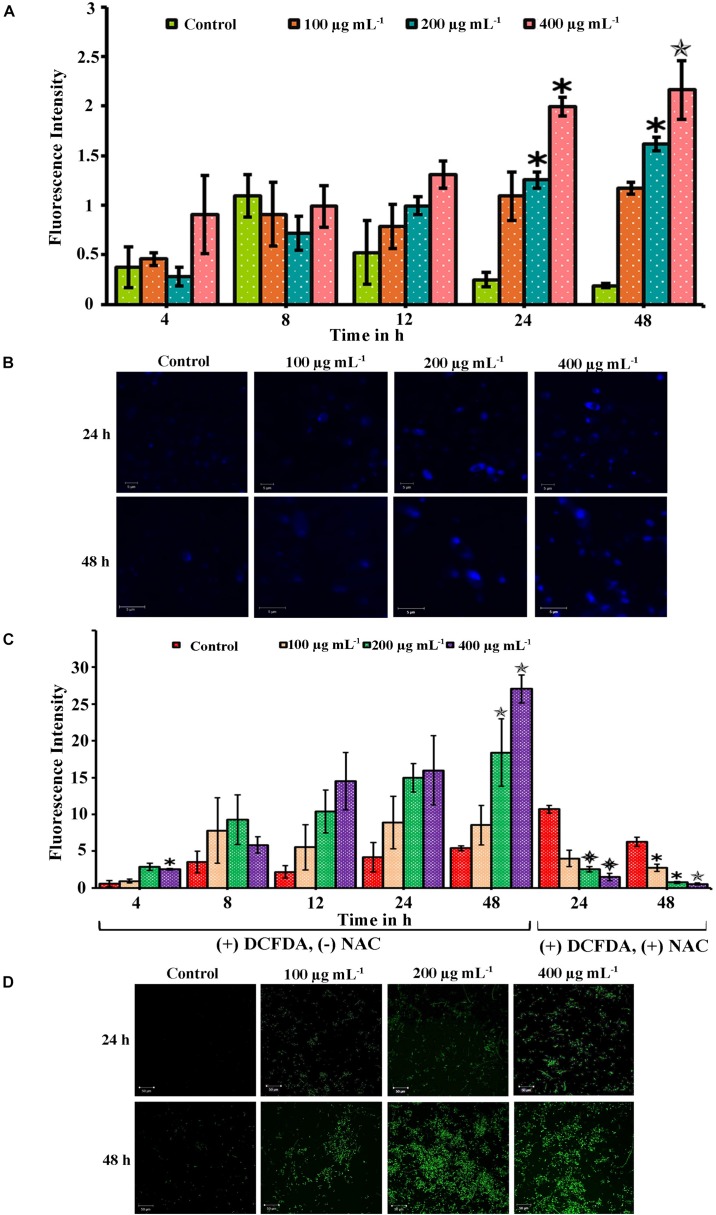
Palmitic acid induced apoptosis and ROS associated with DNA damage in *C. tropicalis*. **(A)** Efficacy of PA inducing apoptosis in *C. tropicalis* at different concentrations (0, 100, 200, and 400 μg mL^−1^) and time points (4–48 h) which was quantified using propidium iodide. **(B)** DNA damage induced by PA at different concentrations were assessed by DAPI staining and visualized in CLSM. **(C)** Extent of ROS produced by PA in *C. tropicalis* after 24 and 48 h treatment was quantified by DCFDA and addition of 10 mM NAC reversed the ROS production in *C. tropicalis.* **(D)** Also, the induction of ROS PA in *C. tropicalis* was visualized in CLSM by DCFDA stain. The values are expressed as means and error bars indicate standard deviation of triplicates (*n* = 6). ✽*p* < 0.05, ✯*p* < 0.01, and 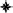
*p* < 0.001, significantly different when compared with control.

When a compound inhibits the growth of the cells, it would induce apoptosis mediated cell death. The apoptosis in the cells was quantified using PI which is impermeable in live cells. The apoptosis in PA-treated cells at different time points (4, 8, 12, 24, and 48 h) was quantified by measuring the fluorescence intensity of PI. The absorbance level is proportional to the number of dead cells in the cell suspension. Compared to control, the absorbance was not increased in 4 and 8 h PA-treated cells (Figure 4A). But in 24 and 48 h treated cells, the absorbance of PI was greater and significantly increased in 200 (*p* < 0.05 and *p* < 0.05, respectively) and 400 μg mL^−1^ (*p* < 0.05 and *p* < 0.01, respectively). Thus, the number of dead cells increased with the increasing concentration gradient of PA (Figure 4A).

### Assessment of DNA Damage by DAPI

Scanning electron microscope analysis confirmed morphological variation in *C. tropicalis* caused by PA and hence the PA induced DNA damage was evaluated by DAPI staining. The elevated fluorescence level was observed in 48 h and 24 h PA-treated *C. tropicalis* at 200 and 400 μg mL^−1^. This showed that PA induced apoptosis by mediating DNA fragmentation and condensed chromatin in *C. tropicalis*. Besides, the intense fluorescence was not inferred in control cells on both time points (Figure 4B).

### PA Triggers ROS Generation in *C. tropicalis*

The extrinsic induction of ROS plays a significant role in apoptosis mediated cell death. This study assessed the qualitative and quantitative analysis of ROS generation in PA-treated *C. tropicalis* by DCF-DA stain. Initially we determined the ROS levels in PA-treated *C. tropicalis* cells at different time points (24 and 48 h) using the ROS specific fluorescent stain DCF-DA. CLSM images revealed the generation of DCF fluorescence initiated at 24 h of PA treatment and it remarkably spiked-up at 48 h time point, with greater generation of ROS at 200 and 400 μg mL^−1^ of PA (Figure 4D). ROS produced by the cells was directly proportional to the extent DCFDA to DCF conversion. Also, the effect of PA in induction of ROS was quantitatively measured at different time points (4, 8, 12, 24, and 48 h). Upon 48 h treatment, PA significantly induced ROS at 200 (*p* < 0.01) and 400 μg mL^−1^ (*p* < 0.01). However, the presence of NAC (ROS scavenger) along with PA significantly revoked the PA induced DNA damage and ROS production in *C. tropicalis* on both 24 h (*p* < 0.001 at 200 and 400 μg mL^−1^) and 48 h (*p* < 0.05 at 100 and 200 μg mL^−1^; *p* < 0.01 at 400 μg mL^−1^) treatment (Figure 4C).

### PA Induced Mitochondrial Dysfunction Mediated Apoptosis

**FIGURE 5:**
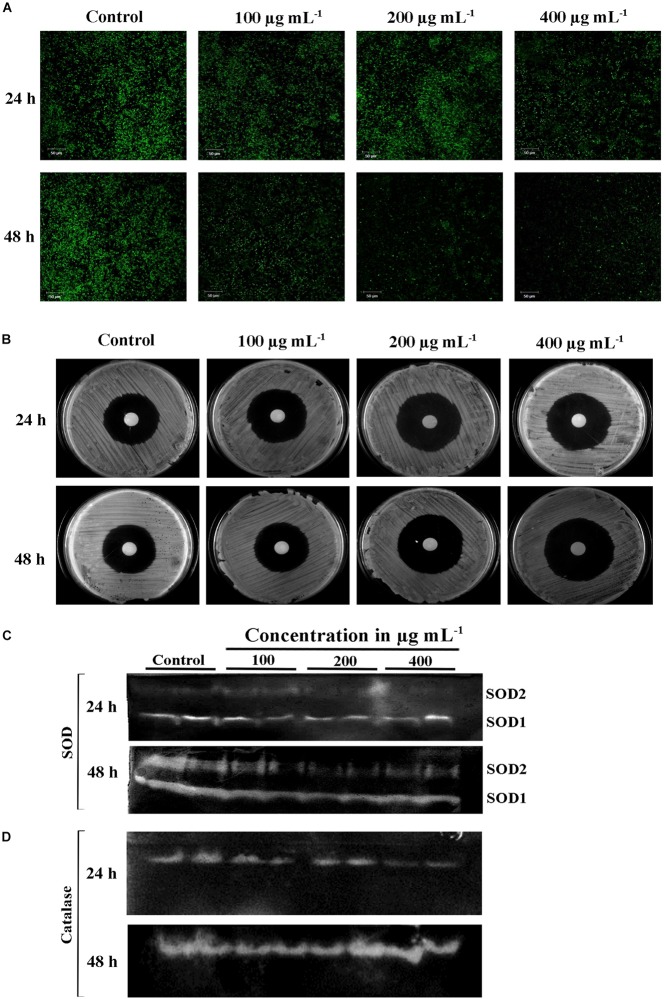
**(A)** Loss of mitochondrial membrane potential (ΔΨm) in *C. tropicalis* at different time points (24 and 48 h) and concentrations (100, 200, and 400 μg mL^−1^) of PA as imaged in CLSM by Rhodamine 123 staining. Presence of PA mediated loss of mitochondrial membrane permeability and induced apoptosis in *C. tropicalis.* **(B)** Representative SDA agar plates depicting the effect of PA on the sensitivity of *C. tropicalis* to H_2_O_2_. Native PAGE revealing expression of superoxide dismutase (SOD) **(C)** and catalase **(D)** in PA (0, 100, 200, and 400 μg mL^−1^) treated *C. tropicalis* after 24 and 48 h.

Mitochondrial membrane potential (ΔΨ m) is important for normal cellular functions such as protein and ATP synthesis (Ott et al., 2007). The intrinsic apoptotic cell death happens during loss of ΔΨ m (Henry-Mowatt et al., 2004). Hence, this study investigated the PA-induced apoptosis mediated by mitochondrial damage using mitochondrial specific probe Rhodamine 123. The stain binds to the active mitochondria and the fluorescence rate was higher in untreated *C. tropicalis* on both 24 and 48 h time points. The fluorescence intensity was decreased with increasing concentrations of PA-treated *C. tropicalis* (Figure 5A). These results clearly indicated that loss of ΔΨ m was associated with PA induced apoptosis thereby activating cell death in *C. tropicalis.*

### Effect of PA on H_2_O_2_ Sensitivity, Catalase, and Superoxide Dismutase *C. tropicalis*

Eukaryotic cell contains antioxidant enzymes such as superoxide dismutase (SOD), catalases and peroxidases that regulates intrinsic ROS-damaging effects. The elevated expression of these antioxidant enzymes would increase the antifungal resistance in *C. albicans* (Seneviratne et al., 2008). We investigated the effect of PA on 24 and 48 h treated *C. tropicalis* catalase and SOD. Briefly, the intracellular proteins of untreated and 24 and 48 h PA-treated *C. tropicalis* at different concentrations were isolated and resolved in native PAGE. PA with increasing concentrations did not affect the *C. tropicalis* catalase on both the time points (Figure 5D). Similarly, PA did not show any impact on *C. tropicalis* SOD1 and SOD2 on 24 h treatment. But upon 48 h of PA treatment, the expression levels of both SODs were reduced with increasing concentrations of PA (Figure 5C). The repression of SOD sensitizes the cells to the oxidative stress and the effect was examined by H_2_O_2_ sensitivity assay. The zone of clearance around the disc signifies the H_2_O_2_ induced oxidative stress in *C. tropicalis.* In 24 h PA-treated *C. tropicalis*, the zone of clearance was similar to control cells (Figure 5B). As expected, the zone was greater in 200 and 400 μg mL^−1^ PA-treated *C. tropicalis* upon 48 h of treatment.

### PA Reduced Cell Surface Hydrophobicity of *C. tropicalis*

**FIGURE 6:**
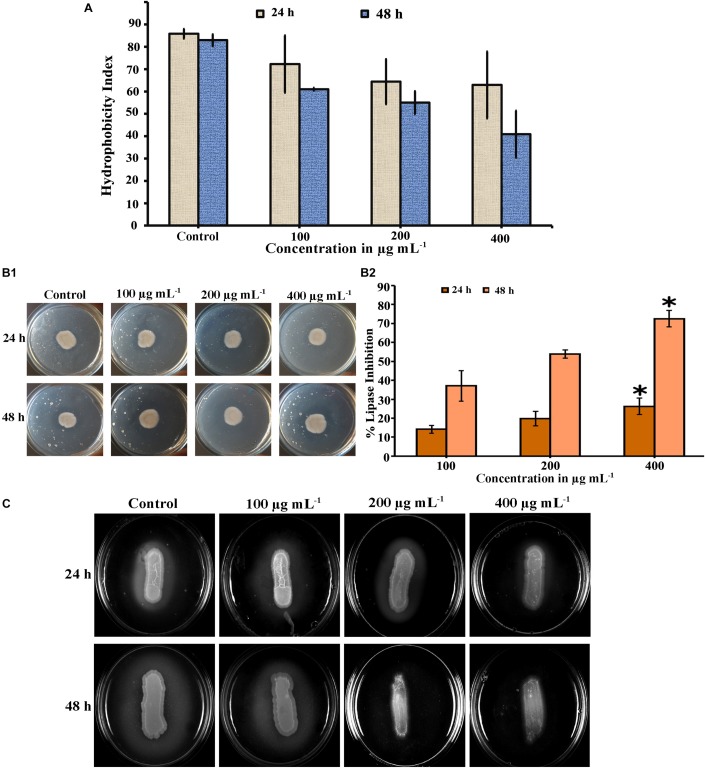
Inhibitory effect of PA on virulence factors of *C. tropicalis*. **(A)** Bar graph showing the reduction of cell surface hydrophobicity in PA-treated (100, 200, and 400 μg mL^−1^) *C. tropicalis* after 24 h and 48 h treatment. **(B)** Effect of PA at different concentrations (100–400 μg mL^−1^) on *C. tropicalis* lipase measured **(i)** quantitatively and **(ii)** qualitatively after 24 and 48 h of treatment. **(C)** Effect of PA on the extracellular protease production of *C. tropicalis* on BSA agar medium. The values are expressed as means and error bars indicate standard deviation of triplicates (*n* = 6). ✽*p* < 0.05, ✯*p* < 0.01 and 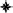
*p* < 0.001, significantly different when compared with control.

Cell surface hydrophobicity (CSH) is a putative virulence attribute in *Candida* spp. on biofilm formation and the effect of PA on CSH of *C. tropicalis* was evaluated by MATH assay. The hydrophobic cells possesses greater affinity toward non-polar solvents. After incubation, the PA-treated and untreated cells were vortexed with toluene and hydrophobicity index was calculated. Results showed that, the affinity of cells to toluene was higher in untreated *C. tropicalis* and the affinity was reduced in 24 h PA-treated cells with increasing concentrations (Figure 6A). But upon 48 h PA treatment, cell affinity toward the solvent was reduced to 40.2% in 400 μg mL^−1^ of PA compared to control (82.95%) (Figure 6A).

### Effect of PA on *C. tropicalis* Extracellular Lipase Production

The lipase activity of untreated and PA-treated *C. tropicalis* was measured quantitatively by using P-Nitro Phenol Palmitate as a substrate. Upon 24 h treatment, PA inhibited lipase at 100 and 200 μg mL^−1^ and significantly got reduced at 400 μg mL^−1^ (*p* < 0.05) (Figure 6B (ii)). Similarly after 48 h treatment, lipase production was found to be decreased in a concentration dependent manner and reduced at 200 and 400 μg mL^−1^ with percentage inhibition of 53.83 and 72.55% (*p* < 0.05), respectively. In addition, the lipase activity was qualitatively measured by tributyrin substrate in solid spider agar medium, resulting in zone of clearance around the growth as shown in Figure 6B (i). The lipase activity in 24 h PA-treated *C. tropicalis* was as similar to control and slight inhibition was observed at 400 μg mL^−1^. But in case of 48 h treated cells, the phospholipase activity was inhibited with increasing in the concentration of PA and significant reduction was observed at 200 and 400 μg mL^−1^.

### Qualitative Analysis of *C. tropicalis* Protease

The effect of PA on *C. tropicalis* protease was examined in BSA solid medium. In this, protease cleaves BSA (substrate) thereby forming zone of precipitation around the cells. In untreated 24 and 48 h cells, a thick zone of precipitation was observed. Upon 24 h PA treatment, the precipitation zone decreased with increasing concentrations and in case of 48 h treatment, the zone was completely inhibited at 200 and 400 μg mL^−1^ PA-treated *C. tropicalis* (Figure 6C).

### Ergosterol Biosynthesis of PA-Treated *C. tropicalis*

**FIGURE 7:**
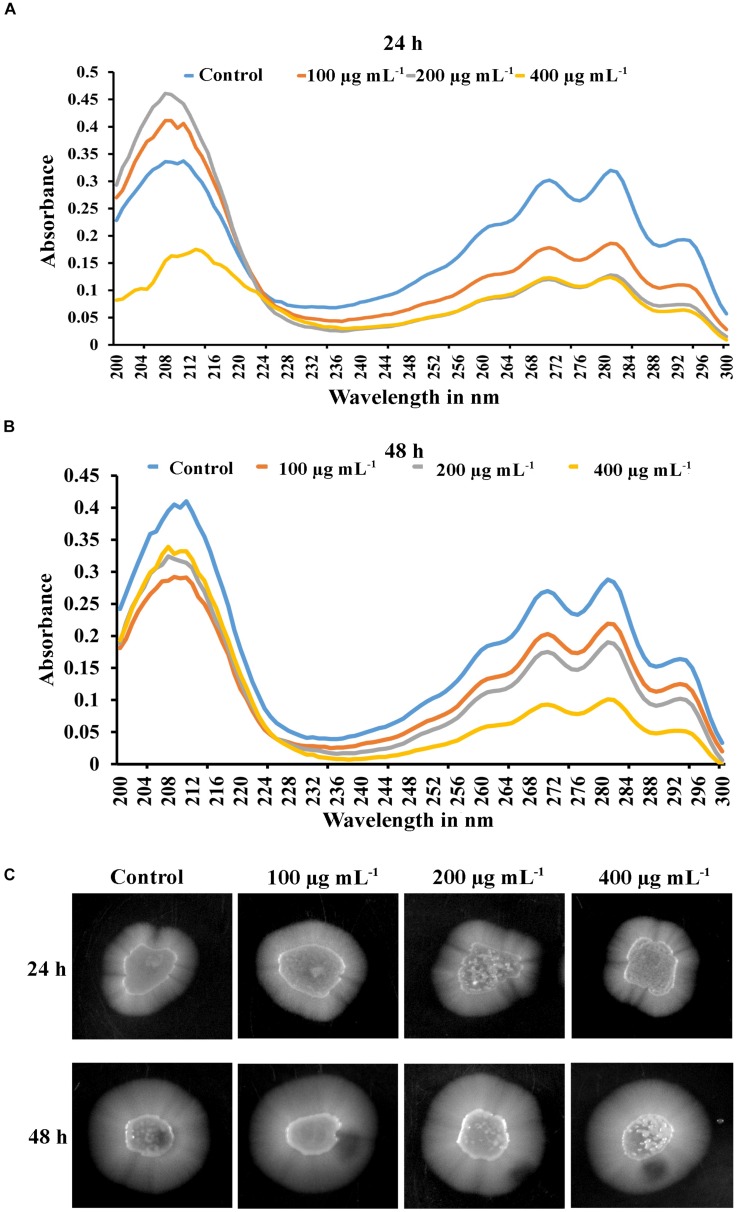
Inhibitory effect of PA (100, 200, and 400 μg mL^−1^) on ergosterol biosynthesis of *C. tropicalis* after 24 h **(A)** and 48 h **(B)** treatment. Ergosterol was extracted by n-heptane and unique sterol spectral profiles of *C. albicans* was measured in the range between 240 and 300 nm. **(C)** PA induced *C. tropicalis* hyphae on spider agar medium with increasing concentrations on both the time points.

The efficacy of PA was investigated on *C. tropicalis* ergosterol biosynthesis at different time points and concentrations. Total ergosterol was isolated from untreated and PA-treated cells by alcoholic KOH followed by extraction with n-heptane. The ergosterol content was measured by UV-spectrophotometer through a unique spectral peak between 240 and 300 nm. PA at 100 and 200 μg mL^−1^ reduced ergosterol production significantly in both the time points when compared to control (Figures 7A,B). Interestingly, ergosterol level of 24 h PA-treated *C. tropicalis* at 400 μg mL^−1^ was similar to 200 μg mL^−1^ (Figure 7A). However, in 48 h treated *C. tropicalis* the ergosterol level in 400 μg mL^−1^ of PA was reduced further to the previous concentration (Figure 7B).

### PA Induces *C. tropicalis* Hyphal Formation

Hyphal formation is one of the virulence factors along with lipase, protease, ergosterol and biofilm in *Candida* spp. The effect of PA was investigated in selective spider medium for hyphal formation in *C. tropicalis*. After treatment with PA on 12 h grown *C. tropicalis* at different concentrations in two different time points such as 24 h and 48 h, the cells were spotted on spider agar medium. Interestingly, PA did not inhibit the *C. tropicalis* hyphae and the level of hyphal formation was similar to control upon 24 h PA treatment. On the contrary, PA at increasing concentrations induced the hyphae in *C. tropicalis* upon 48 h of treatment (Figure 7C). Hence it is obvious that PA did not impact the redevelopment of hyphae but induce the dimorphism with increasing concentrations.

### Validation of Anti-virulence Aspects of *C. tropicalis* by qPCR

Our previous experiments substantiated that PA at 200 μg mL^−1^ upon 48 h effectively reduced mature biofilm, proteases, ergosterol and lipases but failed to inhibit *C. tropicalis* hyphae. Hence, the effect of PA at the above-mentioned concentration and time point was evaluated on *C. tropicalis* virulence genes such as *ERG11, NRG1, TUP1, UME6, HWP1 and EFG* by qPCR. Result showed that PA downregulated the *ERG11* (gene encodes for Lanosterol 14-alpha demethylase) of *C. tropicalis.* Besides, *HWP1* and *EFG1* (gene encodes for Hyphal formation) were overexpressed in PA-treated cells. Interestingly, negative transcription regulators of hyphae such as *NRG1* and *TUP1* were negatively regulated and *UME6*, positive transcription regulator of hyphae was upregulated.

## Discussion

Candidiasis is an infection caused by *Candida* spp. in clinically associated and system specific infections. The occurrence of candida infection on blood stream is known as candidemia. Globally, *Candida* species are the fourth most common pathogen causing Candidemia with the mortality of 38% - 61% (Gudlaugsson et al., 2003) with *C. albicans* at the top. The prevalence of NCAC in candidemia is greater than *C. albicans* with higher mortality in clinically infected immunocompetent patients (Dimopoulos et al., 2008). Mainly in India, the morbidity rate of *C. tropicalis* infection is more prevalent in blood stream infections with 35.3% followed by *C. albicans* (21.5%), *C. parapsilosis* (20%), *C. glabrata* (17.5%), *C. Krusei* (3.3%), *C. haemulonii* (1.5%) and *C. guilliermondii* (1%) (Xess et al., 2007; Chakrabarti et al., 2015). Clinically, the pathogenicity of *C. tropicalis* is more severe than *C. albicans* in oncology patients and neonatal infections (Kothavade et al., 2010). Several virulence factors of *C. tropicalis* such as biofilm and hyphal formation, sterol synthesis, production of hydrolytic enzymes are responsible for invasive infections in immunocompromised patients.

Biofilms are formed by well-structured communication of cells that possess the capacity to penetrate human tissues and thereby causing infections at various sites of the human body (Costerton et al., 1999). The extensive biofilm and hydrolytic enzyme productions in clinically isolated *Candida* spp. makes them more virulent and the longer colonization increases probability of hematogenous infections (Gokce et al., 2007). Also, the adhesion and biofilm in *C. albicans* are the important factors for surface attachment in dental implants (Noumi et al., 2010). In *C. albicans*, hydrolytic enzymes are important for the host-cell membrane damage that promotes invasion and colonization (Monroy-Pérez et al., 2016). The dense biofilm matrices resists the passage of conventional antifungal drugs thereby becoming ineffective (Taff et al., 2013). The compounds derived from natural sources could be a suitable alternative for overcoming antifungal limitations (Rates, 2001), and these compounds are known to have the anti-inflammatory and antimicrobial properties (Ahmad et al., 2014). The broth dilution study and spot assay revealed that PA inhibited 15.23% of growth at 500 μg mL^−1^. The growth was reduced further at 1 mg mL^−1^ with 92.00% of inhibition and reduces the visibile growth of *C. tropicalis*. This study unveiled the MIC of PA against *C. tropicalis* was 1 mg mL^−1^.

Further, the study investigated the antibiofilm activity of Palmitic acid against *C. tropicalis* and other candida spp. such as *C. albicans* and *C. glabrata*. PA inhibited 50% of *C. tropicalis* biofilm even at 100 μg mL^−1^ but failed in case of *C. albicans* and *C. glabrata* biofilm. The dose dependent examination of PA against *C. tropicalis* biofilm revealed the maximum inhibition at 200 μg mL^−1^ and was taken as BIC of PA. Furthermore, the effect of PA on *C. tropicalis* growth was tested using metabolic activity, growth curve and cell viability assays which showed that the PA at BIC showed up a negative regulation of growth and the inhibition was higher at 2× and 4× BIC of PA. *Candida* species produces quorum sensing molecules such as farnesol, tyrosol and farnesoic acid for the maintenance of cellular aggregation for forming biofilms. The extrinsic addition of farnesol inhibits growth mediated biofilm formation of *C. albicans* yeast cells (Uppuluri et al., 2007).

The communication of yeast cells through quorum sensing molecules leads to the cell pile-up thereby forming mature biofilm (Ramage et al., 2002). Hence, the effect of PA on *C. tropicalis* 1, 2, 4, and 7 days matured biofilm was examined at different time points (12, 24, and 48 h). PA at 200 and 400 μg mL^−1^ inhibited more than 50% of 1 and 2 days matured biofilm upon 48 h of treatment and in case of 4 and 7 days matured biofilm, PA was found to inhibit 35.49% of biofilm (Figure 2A). The optical and confocal micrographs of the time killing assay reveals that PA could effectively reduce the biofilm density and thickness upon 48 h of treatment at 200 and 400 μg mL^−1^ (Figures 2B,C). The heterogeneous matrix of mature biofilm retards the penetration of conventional antifungals (Al-Fattani and Douglas, 2004). The potency of PA on *C. tropicalis* mature biofilm formation was higher with increasing concentrations and treatment times. The microbial biofilms are more resistant to antibiotics than early biofilm due to the excessive EPS production (Bowler et al., 2012). However, PA was effective against both early and mature *C. tropicalis* biofilm.

Colony forming unit and Live-dead staining assay showed that the number of dead cells were higher in PA-treated *C. tropicalis* with increasing concentrations and time points. Also, SEM analysis revealed a shrinkage in morphology of 48 h PA-treated *C. tropicalis* at 200 and 400 μg mL^−1^. Hence, it is construed that the ability of PA on *C. tropicalis* mature biofilm disruption could be due to the compound intervention on cellular growth (Figures 3A–C). PA > 1 mg mL^−1^ increases DNA fragmentation in rat pancreatic cells and the subsequent induction of apoptosis creates a damaging effect on β cell mass (Maedler et al., 2001). In this study, the rate of apoptosis was quantified in PA-treated *C. tropicalis* by propidium iodide and the dead cells were higher in 200 and 400 μg mL^−1^ upon 48 h treatment (Figure 4A). Similarly, PA generated DNA damage in *C. tropicalis* to a greater extent at the above-mentioned concentrations and time point.

Ceramide is a part of sphingolipid pathway and acts as an activator of apoptosis cascade due to the cellular stress (Haimovitz-Friedman et al., 1997). But PA generates oxidative stress mediated apoptosis through non-ceramide pathway in Chinese Hamster Ovary (CHO) cells (Listenberger et al., 2001). In *C. tropicalis*, the level of ROS was higher even in 200 and 400 μg mL^−1^ PA-treated cells and the presence of NAC scavenged the ROS generation in PA-treated cells. The excessive generation of ROS creates abnormal mitochondrial permeability. Hence, rhodamine 123 was used to measure the mitochondrial membrane potential (ΔΨ m) and the membrane potential of *C. tropicalis* was reduced with increasing concentrations of PA after 48 h treatment (Figure 5A). Collectively, these results confirmed that the excessive accumulation of ROS and DNA fragmentation in PA-treated cells could contribute to the loss of mitochondrial membrane potential (ΔΨ m) in *C. tropicalis*.

In normal cellular metabolism, balancing of redox homeostasis ensures the survival of cells under oxidative stress conditions. The regulation of antioxidant enzymes, such as superoxide dismutases (SODs), catalases, catalase-peroxidases and peroxiredoxins would result in elevated levels of ROS (Aguirre et al., 2005). SODs are crucial for *Candida* spp. to shield itself from the oxidative mediated cell death (Gleason et al., 2014). Native PAGE revealed that PA regulated SOD2 on both the time points and the reduction of SOD2 sensitized *C. tropicalis* to H_2_O_2_ stress. Surprisingly, PA did not inhibit the catalase activity even at 400 μg mL^−1^.

Clinically, the surface chemistry of medical implants which are more hydrophobic, would increase the bioavailability of devices (Tang et al., 2008). But the hydrophobic nature of cell membrane in pathogens prompts to interact with implants surface and forms biofilm. The treatment of PA reduced the cell surface hydrophobicity of *C. tropicalis* at both the time points (24 and 48 h) and concentrations (100, 200, and 400 μg mL^−1^). Also, lipases and proteases are considered as major virulence factors of *C. tropicalis*. These hydrolytic enzymes contribute to the tissue invasion, colonization and morphological switching in *Candida* species (Park et al., 2013). Previously, myristic acid from *Myristica fragrans* was shown to inhibit lipase and protease by regulating the putative lipase (ATG15) and Candidapepsin (SAP6), respectively (Prasath et al., 2019). In this study, PA at 200 μg mL^−1^ significantly reduced both lipase and protease after 48 h of treatment but the reduction was not detected in 24 h treatment.

Ergosterol is one of the predominant virulence factors found in eukaryotic fungi. The sterol is an essential component for structural organization and function of the plasma membrane in *Candida* species (Lv et al., 2016). In the present study, PA at both the time points reduced the ergosterol content of *C. tropicalis* and significantly inhibited at 200 μg mL^−1^. Most of the antifungals such as azole drugs, nystatin and amphotericin BB targets ergosterol biosynthesis pathway. The prolonged exposure of azole drugs induces expression of Lanosterol α14 - Demethylase (*ERG11*) in *C. albicans* and contributes to the development of resistance against azole drugs (Henry et al., 2000; Song et al., 2004). Hence, the differential expression of *ERG11* in control and 48 h PA-treated *C. tropicalis* at 200 μg mL^−1^ was evaluated by qPCR. The result showed that the expression of *ERG11* was downregulated in PA-treated *C. tropicalis*. Azoles interrupt the ergosterol pathway by interacting with heme group on the active site of lanosterol demethylase (Ji et al., 2000). Mutations in ERG11 plays a major role in development of resistance against antifungals (Xiang et al., 2013). PA effectively downregulated the *ERG11* thereby dwindling the resistance development in *C. tropicalis*.

**FIGURE 8:**
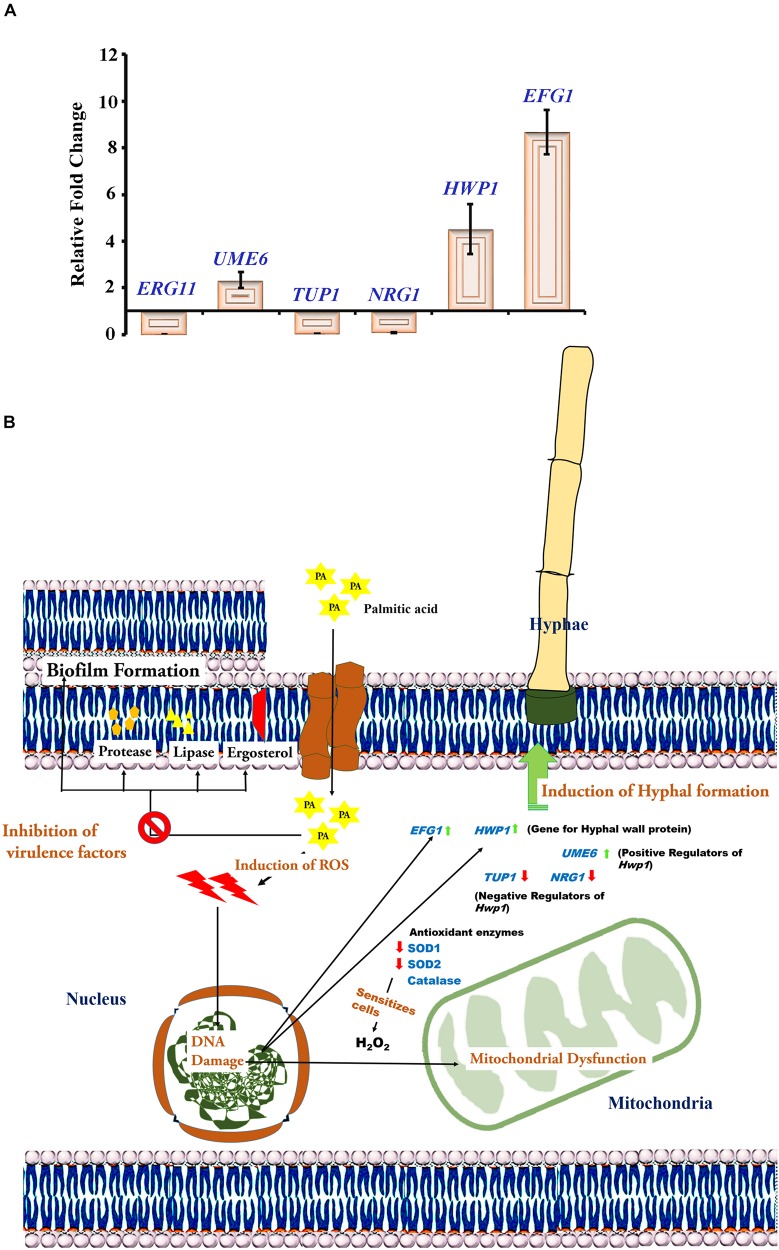
**(A)** Gene expression analysis untreated and PA-treated of *C. tropicalis* at 200 μg mL^−1^ after 48 h. Effect of PA on the differential expression of *ERG11*, *TUP1, NRG1, HWP1, UME6* and *EFG1* using qPCR. The values are expressed as means and error bars indicate standard deviation. **(B)** Molecular Mechanisms of PA at 200 μg mL^−1^ after 48 h inhibiting specific virulence factors of *C. albicans* such as mature biofilm, ergosterol, lipase, protease and cell surface hydrophobicity but induced hyphal formation.

Yeast – hyphal transition occurs in dimorphic fungus based on the host- pathogen interactions and tissue invasion (Li and Nielsen, 2017). Besides, the filamentation assay revealed that PA induced the hyphal elongation in *C. tropicalis* with increasing concentration after 24 and 48 h treatment (Figure 7C). Hyphal wall protein *(HWP1)* is highly expressive in yeast – hyphal transition in dimorphic *Candida* species (Nobile et al., 2008). TUP1 is the transcriptional repressor of filamentous growth and the deletion of the TUP1 gene causes overexpression of hyphae in *C. albicans* (Braun and Johnson, 1997). Also, *NRG1* regulates the hyphal formation and acts as a transcriptional repressor along with *TUP1* and *RFG1* (Braun et al., 2001). The result of filamentation assay was reflected in qPCR with upregulation of *HWP1* and downregulation of *TUP1* and *NRG1* in PA-treated *C. tropicalis* (Figure 8A). However, *UME6*, a positive regulator of hyphal elongation and specific gene for germ tube formation was upregulated. In *C. albicans*, *HWP1* mutant influences on host-pathogen interactions and pathogen morphology with the extent of biofilm production remains unaffected (Orsi et al., 2014). This proved that *Candida* species biofilm and hyphal formation works on different mechanisms and the efficacy of the bioactive(s) on pathogens’ virulence would differ from one factor to another.

Enhanced filamentous growth (EFG1) permits hyphal morphogenesis in *C. albicans* and independent of *TUP1* regulatory mechanism for filamentation (Braun and Johnson, 2000). Similar to *HWP1*, the expression of *EFG1* was also upregulated in PA-treated *C. tropicalis* (Figure 8A). Secreted aspartyl proteases (SAP4-6) are abundant in hyphal cells of *Candida* species. But *SAP4-6* mutants exhibit the filamentation with less invasiveness and the expression of *SAP4-6* is drastically increased in *EFG1* mutants (Felk et al., 2002). Similarly, significant reduction of protease at 200 μg mL^−1^ PA-treated *C. tropicalis* after 48 h of treatment was noticed in the present study (Figure 6C). In *C. albicans*, the extent of hyphal formation is associated with increasing ROS generation in germinating cells (Schroter et al., 2000). Many antifungal agents cause apoptosis through ROS generation in cell death mechanisms against *Candida* infections (Peralta et al., 2015). Curcumin, a well-known potential bioactive exhibits ROS mediated inhibition of biofilm in *C. albicans* but reduces hyphae through *TUP1* repressor pathway (Sharma et al., 2010). Thus, it is predicted that PA reduces virulence factors of *C. tropicalis* through ROS mediated apoptosis but induced hyphal formation. The molecular mechanisms of 48 h PA-treated at 200 μg mL^−1^ targeting specific virulence aspects of *C. tropicalis* is presented in Figure 8B.

## Conclusion

The aggregation of *C. tropicalis* biofilm cells enhances their capacity to evade the host defense and to resist conventional antibiotics. The cell-aggregation of biofilm community in close proximity displays similar transcriptomic pattern especially in genes coding for antibiotic resistance (Bjarnsholt et al., 2013). This kind of community is well established in mature biofilm by means of robust exopolymers. Also, the failure of conventional antifungal arises due to the restricted penetration in mature biofilm. The current study explicated that PA at 200 μg mL^−1^ induced ROS and apoptosis in *C. tropicalis* thereby reducing various virulence factors such as mature biofilm, cell surface hydrophobicity, ergosterol, lipases and proteases after 48 h of treatment. Besides, PA induced hyphal formation in *C. tropicalis* with increasing concentrations. In addition, gene expression analysis revealed that PA downregulated *ERG11* and reduced the probability of developing antifungal resistance. Further, PA upregulated genes responsible for hyphal formation, i.e., *EFG1* and *HWP1* that correlates the filamentation assay. Thus, the efficacy of PA targeting biofilm associated virulence, could be used in the synergistic strategy that provides improved therapeutic effects against NCAC infections.

## Data Availability Statement

All datasets generated for this study are included in the article/supplementary material.

## Author Contributions

SP conceived and supervised all the experiments and KP designed the work flow. KP, HT, and MK performed the experiments and data analyses. KP wrote the main manuscript text and prepared all the figures. All authors reviewed the manuscript.

## Conflict of Interest

The authors declare that the research was conducted in the absence of any commercial or financial relationships that could be construed as a potential conflict of interest.
